# City level water withdrawal and scarcity accounts of China

**DOI:** 10.1038/s41597-024-03115-4

**Published:** 2024-05-03

**Authors:** Zongyong Zhang, Yuli Shan, Dandan Zhao, Martin R. Tillotson, Bofeng Cai, Xian Li, Heran Zheng, Cunxue Zhao, Dabo Guan, Junguo Liu, Yu Hao

**Affiliations:** 1 School of Management and Economics, Beijing Institute of Technology, Beijing, 100081 China; 2 School of Environmental Science and Engineering, Southern University of Science and Technology, Shenzhen, 518055 China; 3 Water Security Research Centre, School of International Development, University of East Anglia, Norwich, NR4 7TJ UK; 4 School of Geography, Earth and Environmental Sciences, University of Birmingham, Birmingham, B15 2TT UK; 5 Water & Development Research Group, Department of Built Environment, Aalto University, Espoo, 00076 Finland; 6 School of Civil Engineering, University of Leeds, Woodhouse Lane, Leeds, LS2 9JT UK; 7 Centre for Climate and Environmental Policy, Chinese Academy for Environmental Planning, Beijing, 100012 China; 8 Department of Statistics and Data Science, Southern University of Science and Technology, Shenzhen, 518055 China; 9 The Bartlett School of Sustainable Construction, University College London, London, WC1H 0QB UK; 10 Business School, Hohai University, Nanjing, 211100 China; 11 Department of Earth System Science, Tsinghua University, Beijing, 100084 China; 12 The Bartlett School of Construction and Project Management, University College London, London, WC1E 7HB UK; 13 Center for Energy and Environmental Policy Research, Beijing Institute of Technology, Beijing, 100081 China; 14 Yangtze Delta Region Academy of Beijing Institute of Technology, Jiaxing, 314001 China; 15 Sustainable Development Research Institute for Economy and Society of Beijing, Beijing, 100081 China

**Keywords:** Sustainability, Water resources, Developing world, Interdisciplinary studies

## Abstract

In the context of China’s freshwater crisis high-resolution data are critical for sustainable water management and economic growth. Yet there is a dearth of data on water withdrawal and scarcity regardless of whether total or subsector amount, for prefectural cities. In administrative and territorial scope, we accounted for water withdrawal of all 63 economic-socio-environmental sectors for all 343 prefectural cities in China, based on a general framework and 2015 data. Spatial and economic-sector resolution is improved compared with previous studies by partitioning general sectors into industrial and agricultural sub-sectors. Construction of these datasets was based on selection of 16 driving forces. We connected a size indicator with corresponding water-withdrawal efficiency. We further accounted for total blue-water withdrawal and quantitative water scarcity status. Then we compared different scopes and methods of official accounts and statistics from various water datasets. These disaggregated and complete data could be used in input-output models for municipal design and governmental planning to help gain in-depth insights into subsector water-saving priorities from local economic activities.

## Background & Summary

Faced with a freshwater scarcity crisis^1–3^, China has implemented a nationwide and stringent *‘Three Redlines’* policy regime to 2030^4^. These regulatory constraints were imposed on water withdrawal, even in non-arid areas, to promote sustainable water use and economic development^5,6^. However, as a basic unit to inform water regulation policies, there was a lack of readily available city-level water withdrawal and scarcity data regardless of whether this is total or subsector level^7,8^ (here a ‘city’ is the term used for sub-provincial and prefecture-level administrative units, including leagues, regions and autonomous prefectures; a ‘sector’ is defined according to the latest classifications of national accounting system for economic activities, which can be concordant with international and widely-used classifications i.e., CPC and ISIC^9^). Due to the absence of measured efficiency data i.e., subsector water-use per unit of economic value, it is therefore difficult to quantify water withdrawal or optimize water use efficiency. Thus, accounting for the water withdrawal of different sub-sectors and water scarcity for prefectural cities could help practitioners understand water use and assess water sustainability^10–12^.

Current research is mainly limited to water use by the energy sector, and neglects many other water-consuming sectors. For example, water withdrawal inventories are available for construction of large coal-fired power generation hubs^13^, but wider sectoral and systematic water withdrawal datasets are not. This limitation in statistical and accounting data is long standing, lasting two decades^1,4^. A data gap is in measured water efficiency data i.e., subsector water-use per unit of economic value^14,15^.

According to the literature, high-resolution spatial and economic-sector data are critical for sustainable water management^16,17^, and water withdrawal data are amongst the most sought^18–20^. For example, a recent perspective in *Nature Water*^20^ appealed for open-access availability of China’s water withdrawal data. Current data is typically based on geographic grid units, rather than on an administrative-territory basis^21,22^. For administrative-territory accounts, Hoekstra and Chapagain^23^ estimated national water use of different countries from a production perspective, by introducing agricultural water use efficiency as a factor on water consumption. A few institutions provided national and sectoral water withdrawal data, such as AQUASTAT from the Food and Agriculture Organization (FAO). Nevertheless, (1) this data was too general to be partitioned into more disaggregated prefectural-cities. For prefectural scope, only Zhou *et al*.^24^ could provide total water withdrawal data before 2013 through simulation based on survey and statistical data from the *Ministry of Water Resources*^24^. Yet this data is not fully open, and the data-source information is difficult to review or trace back due to partial disclosure of information and government sensitivity to water issues. This situation has created challenges and difficulties with data comparability, quality and reflection. (2) These data typically treat construction, services and households as a single sector classified as *domestic water use*^25^. This omits water withdrawal information or finer sub-sector difference within construction, services or households. In fact, water withdrawals for construction and services account for an average of about 20% of total domestic water withdrawal^14^.

Internationally speaking, water accounting in China now lags behind other developed countries such as Australia, the U.S. and France^26–28^. Sectoral water accounts have been established in several countries at the national level, e.g., Australia, Denmark, France, the Netherlands, New Zealand, Spain and the U.S. Taking the U.S. and Australia as state-of-the-art examples, considerable fragmentation of water accounting methods in the former resulted in the introduction of the CEO Water Mandate aimed at the ability of companies to measure and communicate water in a consistent manner^9^ (https://seea.un.org/sites/seea.un.org/files/ungc_cwaf_presentation_june_25_2019_0.pdf). The Mandate proposes and promotes the most cutting-edge water topics, such as urban and local water use information disclosure from large companies, water valuation, and return on investment, etc., to improve water resilience. Similar themes are not discussed or progressed in China. Similarly, water accounting in Australia was introduced following the Millennium Drought of 2000, and has become a well-known program for presentation of water-use information. For example, Australia has a water accounting framework in its mining and metal industries. Prior to this, approaches to measuring, monitoring and reporting on water use were often inconsistent between sites within companies or across sectors. To address this inconsistency, a framework was developed by the University of Queensland Sustainable Minerals Institute and, after more than six years of revision, exploration and data accumulation, was adopted as a common industry approach to water accounting. In other words, benchmark water withdrawal efficiency data are given as standard performance, for the framework to be adapted to a range of local contexts. In contrast, water withdrawal statistics in China are patchy, and water data across all sub-sectors at the city level appeared to be relatively insufficient. In summary, there remains a dearth of subsector, open-access and detailed water withdrawal datasets for China. Collation and sharing of such datasets should be encouraged, and publication of subsector water withdrawal and total water scarcity data ought to be the first step in enriching existing knowledge and alleviating water stress at the city level^24,29^.

In order to help alleviate this situation, we accounted for water withdrawal for all 63 economic-socio-environmental sectors in all 343 prefectural cities in China, totaling 21,609 sub-sectors. This was achieved using a previously reported framework^15^, based on 2015 data. Spatial and economic-sector resolution has been improved compared to previous studies^24,30,31^ through partitioning general sectors into 11,152 industrial and 1,715 agricultural sub-sectors. The methodology included selection of 16 driving forces for water withdrawal, and we connected each of 9 size indicators with their respective water-withdrawal efficiencies given different availability of water statistics collected from cities and provinces. The framework thus combines incongruent water-use data into one consolidated information set for a developing country. In particular, industrial water withdrawal efficiency and benchmark performance were obtained from a point-source survey. We used the data of national pollution source census and regular reporting systems of the *Ministry of Ecology and Environment*, covering 161,598 companies from 14,063 sector-city combinations, i.e., 343-prefecture times 41-industrial-sector in China. These disaggregated and complete datasets are transparent, verifiable, and up-to-date for China. We have named our data the ‘Dataset on Water Use of China at the Sub-sector level (DWUCs)’.

**Fig. 1:**
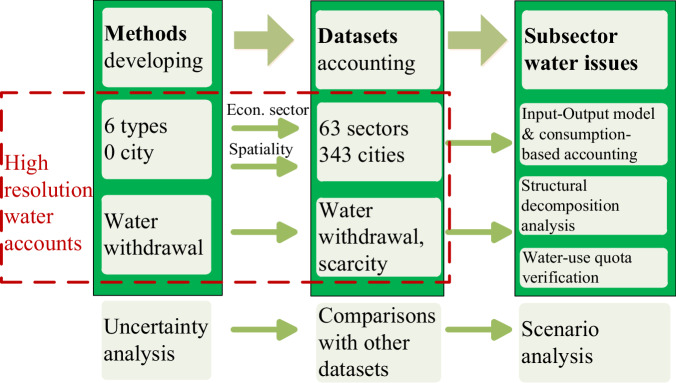
A schematic overview of workflow, including potential applications of DWUCs.

The datasets could be used in Input-output (IO) models (with environmentally extended input-output analysis at the megacity-, province-, and nation-levels)^32^, consumption-based accounting and structural decomposition analyses^33,34^. For example, other publications used a part of DWUCs to gain insights into subsector water-saving priorities through improving low water use efficiency in local economic activities^35^, hence enabling a water-saving society at the city level^36–39^. Apart from subsector datasets, we further accounted total blue-water withdrawal, and quantitative water scarcity status. These data may therefore be used to alleviate water stress^40,41^ (Fig. 1).

## Methods

**Fig. 2:**
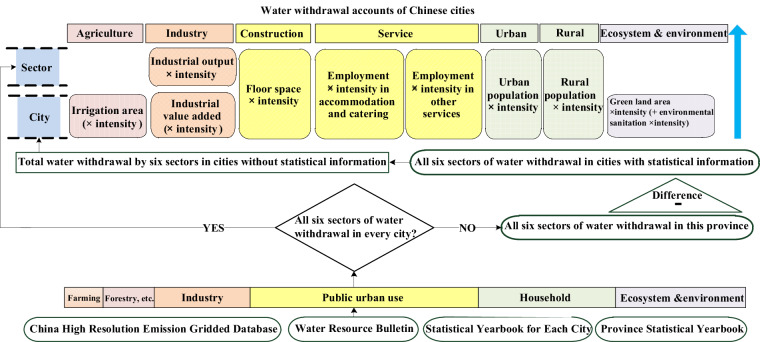
A framework for subsector water withdrawal accounting at the city level. The circled sources at the bottom indicate the primary data inputs used for estimation. This is in furtherance to our previous paper^14^.

The accounting scope and methods are expanded versions of descriptions in our related work (Fig. 2)^15^. In this study, water accounting means statistical estimation of water withdrawal (total and sectoral), and water withdrawal-to-availability ratio (as a measurement of physical and quantitative water scarcity) at the city level.

### Accounting scope

#### Water withdrawal

According to the Intergovernmental Panel on Climate Change (IPCC) administrative and territorial scope^42^, we adopted Scope 1 for city water withdrawal. Scope 1 water withdrawal refers to anthropogenic water withdrawal ‘*taking place within national (including administered) territories and offshore areas* (pageoverview.5)’. In other words, Scope 1 accounts for all types of water withdrawal within a city boundary: farming, forestry, animal husbandry, fisheries, industry, construction, service, household, and ecosystem and environment preservation. There are 63 sectors in total, as listed in Table 1. It is worth noting that the boundary of a ‘city’ spans both rural and urban geographies, which are distinguished from built-up districts indicating only a part of an urban area.

**Table 1:** A list of 63 sectors in this study.

1	Wheat cultivation	33	Smelting & pressing of nonferrous metals
2	Maize cultivation	34	Metal products
3	Vegetables & fruits cultivation	35	General purpose machinery
4	Fiber & bean etc. cultivation	36	Special purpose machinery
5	Rice cultivation	37	Transport equipment
6	Forestry, animal husbandry & fishery	38	Electrical machinery & equipment
7	Coal	39	Communication equipment, computers & other electronic equipment
8	Extraction of petroleum & natural gas	40	Measuring instruments & machinery for cultural activity & office work
9	Ferrous metal ores	41	Other manufacture
10	Nonferrous metal ores	42	Comprehensive utilization of waste resources
11	Nonmetal ores	43	Electric power, steam & hot water
12	Other minerals	44	Gas
13	Processing of food from agricultural products	45	Tap water
14	Foods	46	Construction
15	Liquor, beverage & refined tea	47	Wholesale, retail trade
16	Tobacco	48	Transportation, warehousing & postal industry
17	Textile	49	Accommodation & catering industry
18	Textile wearing apparel & caps	50	Information transfer, computer service & software industry
19	Leather, fur, feather & related products & footwear	51	Financial industry
20	Processing of timber, wood, bamboo, rattan, palm & straw products	52	Real estate
21	Furniture	53	Leasing & business services
22	Paper & paper products	54	Scientific research & technical services
23	Printing, reproduction of recording media	55	Water, environment & public facilities management
24	Culture, education, handicraft, fine arts, sports & entertainment articles	56	Resident services & other services
25	Processing of petroleum, coking & nuclear fuel	57	Education
26	Raw chemical materials & products	58	Health & social work
27	Medicines	59	Culture, sports & entertainment
28	Chemical fibers	60	Public management, social security & social welfare
29	Rubber	61	Urban household
30	Plastics	62	Rural household
31	Nonmetallic mineral products	63	Environment & ecology
32	Smelting & pressing of ferrous metals		

Notes: 1–5 are crop cultivation; 7–12 represent mining and processing; 13–42 are manufacturing; 43–45 are production and supply of electricity, gas and hot water; 47–60 are services; and 61–62 are households. This classification was sourced from the *Industrial Classification for National Economic Activities* promoted by the National Administration for Quality Supervision, Inspection and Quarantine^9^.

Additionally, industrial water withdrawal covers water use of coal-fired and nuclear power plants, but excludes intra-river water use such as for hydro-power generation. Agriculture comprises farming, forestry, animal husbandry and fisheries. For construction, we do not calculate roads or railways. Due to their large scales of engineering, their construction usually could be divided into different segmentations and across different cities. Thus we select ‘floor space of housing’ as a representative type in a diverse construction structure, considering that (1) this indicator is the most reliable to access from statistics; (2) installations for infrastructure, including machines, devices, equipment, process piping etc., are included.

#### Water scarcity

Water scarcity may be indicated by a criticality ratio, calculated as water withdrawal-to-availability. In terms of measuring scarcity, the Falkenmark indicator is a well-known measurement with per capita renewable water resource, nevertheless, it does not reflect the environmental flow requirement^1,43^. The criticality ratio is a simple and classical indicator of blue water (surface fresh water, i.e., water in rivers, lakes and reservoirs) and quantitative scarcity^44,45^ connecting anthropogenic water withdrawal with natural water quantity^46^, and taking into consideration both environmental flows^47,48^ and natural biodiversity^49^. Yet it has thus far not been applied at the city level^1,50–52^ due to water withdrawal data limitations^16^.

### Accounting method

#### Water withdrawal

**(1)** Based on water balances between prefectures and provinces^24^, we followed three specific steps for industrial water withdrawal accounting **(1.1–1.3)**. We realized the city level partition **(1.2)**, and then the sub-sector level **(1.3)**:

For **(1.1)** we compiled industrial water withdrawal for cities in a province from provincial Water Resources Bulletins. There were two cases to consider based on data availability:

*Case 1*) If the Water Resources Bulletin for a province provided industrial water withdrawal for every administrative city these data were compiled for sub-sector partition (section **1.3**). We allocated water withdrawal into each disaggregated sub-sector (see Table 1) for each city.

*Case 2*) If the Water Resources Bulletin did not provide water withdrawal for industrial type for all administrative cities, we collected industrial water withdrawal for each city in their corresponding bulletins. For those cities that did not have these data in their respective bulletins, we used a water mass balance approach to calculate the difference between provincial water withdrawal and the sum of water withdrawal for all cities that did have statistics in their city-level bulletins. In this way we obtained a sum for all cities for which water withdrawal by industrial type was not included in their Water Resources Bulletins.

For **(1.2)**, we allocated the sum of industrial type for those cities without statistics based on two multipliers as driving forces of water withdrawal. We used total industrial value added (*Valueadded*, size indicator) multiplying water withdrawal per value added (*Intensity*_*i*_) in the partition. According to data availability, for cities having data for both water withdrawal per value added and total industrial value added, we immediately obtained:

1 $${W}_{i}=Valueadde{d}_{i}\times Intensit{y}_{i}/\mathop{\sum }\limits_{i=1}^{n}(Intensit{y}_{i}\times Valueadde{d}_{i})$$

2 $$WaterIndu{s}_{i}={W}_{i}\times SU{M}_{n\_cities}$$

where *i* is a city without statistics in the province, and *n* represents the total number of cities without statistics in the same province. This treatment is considered a step beyond previous studies^53^ which assumed industrial water withdrawal per value added was identical among regions. In the case of missing water intensity, we instead used total industrial value added to calculate proportions to disaggregate water withdrawal; we acknowledge this does result in uncertainty.

For **(1.3)**, we used disaggregated industrial output^54^ and water withdrawal per output of each sub-sector (*Intensity*_*i,k*_) to partition total industrial water withdrawal of each city (*WaterIndus*), i.e.:

3 $${W}_{i,k}=Intensit{y}_{i,k}\times Outpu{t}_{i,k}/\mathop{\sum }\limits_{k=7}^{45}(Intensit{y}_{i,k}\times Outpu{t}_{i,k})$$

4 $$Wate{r}_{i,k}={W}_{i,k}\times WaterIndu{s}_{i},k\in [7,45]$$

where *k* represents a sub-sector of city *i*. Overall, we extracted water withdrawal intensities (water withdrawal per industrial output, m^3^ per million US$) of survey companies to use them in subsector estimation.

Similarly, for other types such as agriculture, construction, service, household and environment, we accounted for subsector and total water withdrawal^36^, i. e.:

**(2)** we used irrigation water withdrawal per mu for farmland (1 mu ≈ 667m^2^, and is commonly used by Water Resources Bulletins in provision), and the irrigation area to determine agricultural water use according to data availability: *Case 1)*, for cities with data for both irrigation water withdrawal per mu (*Intensity*) and irrigation area (*Irriareas*), we immediately obtained:

5 $$Wate{r}_{i,1}=Irriarea{s}_{i,1}\ast Intensit{y}_{i,1}$$

And in *Case 2)*, if a city did not provide the irrigation water withdrawal per mu, we used the irrigation area instead:

6 $$Wate{r}_{i,1}=Irriarea{s}_{i,1}/\mathop{\sum }\limits_{i=1}^{j}Irriarea{s}_{i,1}\ast Wate{r}_{j,1}$$

where *j* denotes the number of cities that did not provide figures in their own Water Resources Bulletins, and *Water*_*j*, 1_ represents the sum of agricultural water withdrawal for those cities without statistical information.

**(3)** We utilized the floor space of housing (*Flospac*) and the water withdrawal per unit (*Intensity*) to estimate water withdrawal for construction. For water withdrawal for accommodation and catering, which is usually the largest water user in the service sector, we assumed a positive correlation between water use and the number of employees, and then used employment and water withdrawal per employee (*Intensity*):

7 $$Wate{r}_{i,46}^{{\prime} }=Flospa{c}_{i,46}\ast Intensit{y}_{i,46}$$

8 $$Wate{r}_{i,49}^{{\prime} }=Employmen{t}_{i,49}\ast Intensit{y}_{i,49}$$

9 $$Wate{r}_{i,k}^{{\prime} }=Employmen{t}_{i,k}\ast Intensit{y}_{i,k},k\in [47,48]\cup [50,60]$$

**(4)** We used the rural population (*Popul*, permanent residents) and household water withdrawal per capita in rural areas (*Intensity*) to estimate rural household water withdrawal. The estimation for urban household water withdrawal was similar, i.e.,

10 $$Wate{r}_{i,k}^{{\prime} }=Popu{l}_{i,k}\ast Intensit{y}_{i,k}$$

11 $${W}_{i,k}=Wate{r}_{i,k}^{{\prime} }/\mathop{\sum }\limits_{k=61}^{62}Wate{r}_{i,k},k\in [61,62]$$

For the subsector module we used the proportions of water withdrawals (initial magnitude indicated by *Water’*) in construction, accommodation and catering, and other services to separate urban and public water withdrawal:

12 $$Wate{r}_{i,k}=Wate{r}_{i,k}^{{\prime} }/\mathop{\sum }\limits_{k=46}^{60}Wate{r}_{i,k}^{{\prime} }\ast (Wate{r}_{i,UrbanPublic}),k\in [46,60]$$

13 $$Wate{r}_{i,k}={W}_{i,k}\times Waterhousehol{d}_{i},k\in [61,62]$$

**(5)** We used the area of green land, irrigation volume per area of green land in urban areas (*Intensity* equals 0.0782 cubic meters), environmental sanitation area (*Sanitarea*), and water withdrawal per unit (*Intensity’* equals 0.0265 cubic meters) to estimate ecosystem and environment water withdrawal, i.e.,

14 $$W{{\rm{ater}}}_{i,63}=Greenlan{d}_{i,63}\ast Intensit{y}_{i,63}+Sanitare{a}_{i,63}\ast Intensit{y}_{i,63}^{{\prime} }$$

**Fig. 3:**
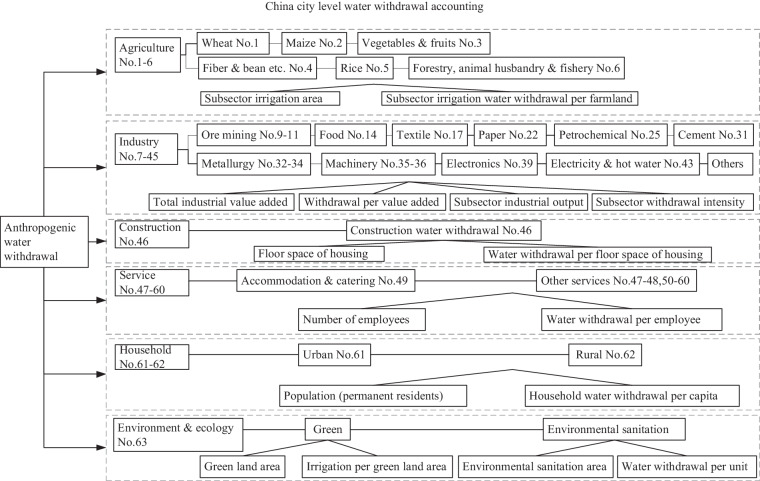
Featured selections of 16 driving forces for water withdrawal datasets.

In summary, a series of socio-economic driving forces were selected and connected to water withdrawal of individual type (Fig. 3). Specifically, there were 9 size indicators: (1) irrigation area; (2) total industrial value added; (3) sectoral industrial output; (4) floor space of housing; (5) number of employees in accommodation & catering; (6) number of employees in other services; (7) permanent resident population (rural and urban, respectively); (8) green land area; and (9) environmental sanitation area, respectively. We supplemented Table 2 to show the indicators and their data sources. These indicators were connected to their respective efficiency. The efficiency indicators were, (1) irrigation water withdrawal per mu for farmland; (2) water withdrawal per industrial value added; (3) disaggregated water withdrawal intensity of each industrial sector; (4) water withdrawal per floor space of housing; (5) water withdrawal per employee in accommodation & catering; (6) water withdrawal per employee in other services; (7) household water withdrawal per capita in rural (and urban) areas; (8) irrigation volume per area of green land in urban areas; and (9) water withdrawal per environmental sanitation area. For example, in service, we used the number of subsector employees rather than value added^24^, considering it to be more reasonable to assume a positive correlation between water use and the number of employees, rather than value added to the economy in the service sector. For household, because urban residents usually use more water per resident than rural residents, how much water a city uses should be determined by not only its absolute population but also its urbanization structure. Thus, it is necessary to combine the population with its respective water withdrawal per resident.

**Table 2:** Driving-force indicators and their source.

Agriculture		Irrigation area ○	○ Province statistical yearbook. Δ City statistical yearbook. ○/Δ Province or city statistical yearbook or internet search. √ Water Resource Bulletins. ※ China High Resolution Emission Gridded Database. X Bulletin of the First Water Resources Census (the 2nd Water Census of Shanghai)). ∪ China City Statistical Yearbook.
		Irrigation water withdrawal per mu of farmland √	
Industry		Total industrial value added ○/Δ	
		Water withdrawal per industrial value added √	
		Sectoral industrial output Δ	
		Disaggregated water withdrawal intensity of each sector ※	
Construction		Floor space of housing ○	
		Water withdrawal per floor space of housing X	
Services	Accommodation & catering	Number of employees in accommodation & catering ∪	
		Water withdrawal per employee in accommodation & catering X	
	Other services	Number of employees ∪	
		Water withdrawal per employee in other services X	
Household	Rural	Rural population (permanent residents) ○	
		Household water withdrawal per capita in rural areas ○/Δ	
	Urban	Urban population (permanent residents) ○	
		Household water withdrawal per capita in urban areas ○/Δ	
Ecosystem & environment		Area of Green land ∪	
		Irrigation volume per area of green land in urban areas X	
		Environmental sanitation area Δ	
		Water withdrawal per unit X	

Note: this is in furtherance to a previous publication^14^.

#### Water scarcity

Referring to previous studies^32,36,51,55,56^, we applied the criticality ratio (%) to measure annual water scarcity in Eq. (15), i.e.:

15 $$Criticality\_rati{o}_{i}=\frac{\mathop{\sum }\limits_{k=1}^{63}Water\_withdrawa{l}_{i,k}}{Water\_availabilit{y}_{i}}$$

where *i* represents a city; *k* is a sector in city *i*; ${\sum }_{k=1}^{63}Waterwithdrawa{l}_{i,k}$ is the total amount of farming, forestry, animal husbandry, fisheries, industry, construction, service, household, and ecosystem and environmental preservation (for detailed description please refer to Table 1).

The higher the criticality ratio the more stress is placed on available water resources from withdrawal, and the greater the occurrence of water scarcity^57,58^. A criticality ratio >40% is generally accepted as high water scarcity status, and over 100% is regarded as extreme water scarcity.

**Fig. 4:**
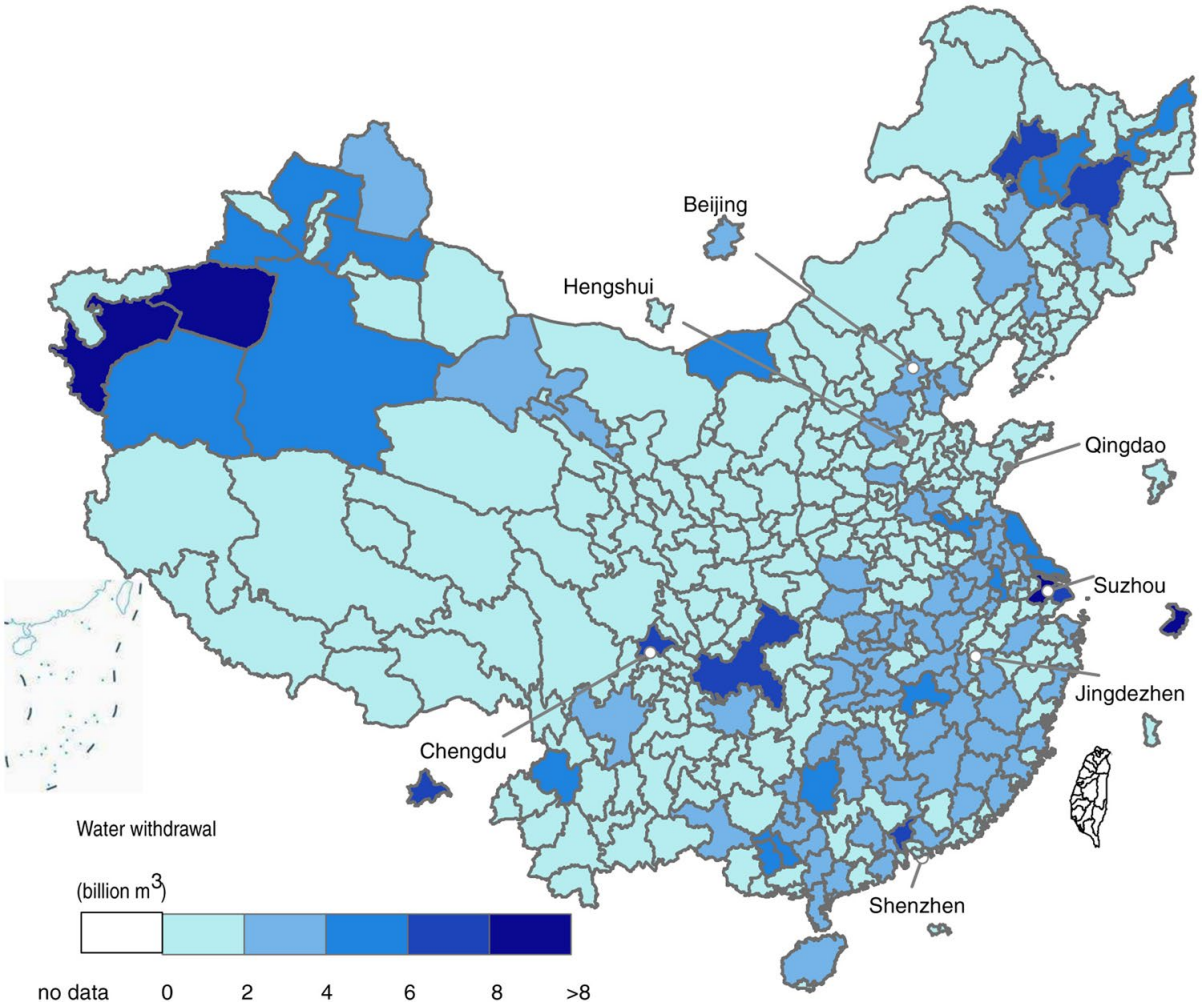
A map of China showing prefectures and water-withdrawals. This is a visual representation of the study region, drawn based on datasets. The labeled cities are briefly discussed in this study.

Cities from Jiangsu, Zhejiang, Guangdong, Anhui, Hainan, Heilongjiang, Tibet, and Jilin provinces were categorized as *Case 2*, with the remaining cities as *Case 1*. In this way we have constructed city-level water withdrawal inventories with 63 sectors (columns) and 343 cities (rows) i.e., 21,609 sub-sectors, total water withdrawal (the 64^th^ column), water availability (the 65^th^ column) and criticality ratio (the 66^th^ column) for China. Of the 343 cities, 272 (representing 88% of China’s population) had available data for industrial subsector accounting, and all 343 were further accounted for total water withdrawal (Fig. 4) and scarcity status.

### Data sources

For 14,063 sub-sectors (343 prefectures and 41 industrial sectors, Table 1), individual intensities (water withdrawal per industrial output, m^3^ per million US$) were derived from the national pollution source census and regular reporting systems of the *Ministry of Ecology and Environment*. A point-sourced census of 2015 covered 161,598 companies in total (approximately 42% of all above designated-size companies in China. An above designated-size company refers to those with annual revenues >US$2.93 million^59^ based on an exchange rate of US$1 = Ұ6.8174). Using this survey we obtained water withdrawal efficiency as benchmark performance; specifically intensity was calculated as the subsector water withdrawal divided by the industrial output of sample companies. In the census and survey, industrial output data were coupled with water withdrawal data of companies. Then these data were aggregated according to sub-sectors to calculate the intensities.

The six types of total water withdrawal, irrigation water withdrawal per mu for farmland, industrial water withdrawal per value added, and water availability were sourced from Water Resources Bulletins at province and city levels^56^. The Bulletins were issued by the *Ministry of Water Resources* and local *Hydrology and Water Resources Investigation Bureaus*, referred to previous studies^32,36,55,56^. Total irrigation water withdrawal for farming in 156 cities, subsector irrigated area and subsector water withdrawal per irrigated area data (m^3^ per m^2^ of 5 main crops i.e., rice cultivation, wheat, maize, vegetables and fruits, fiber and beans etc.) of 343 prefectures were obtained from Zhou *et al*.^24,60^.

Water withdrawal per unit of floor space of completed housing, water withdrawal per capita in representative accommodation and catering, water withdrawal per capita in other services, irrigation water withdrawal per area of green land in urban areas, and water withdrawal per environment and sanitation area were sourced from the Bulletin of 1^st^ Water Resources Census (i. e., 2^nd^ Water Resources Census of Shanghai by the Shanghai Bureau of Statistics and Water Authority). Irrigation area, floor space of housing, rural and urban population (permanent resident) were obtained from provincial Statistical Yearbooks^59,61^. Subsector industrial output, and environmental sanitation area were taken from the Statistical Yearbooks for each city^14,61^. The subsector industrial output data were then proofed and corrected by the authors, according to the China City Statistical Yearbook. We regarded the China City Statistical Yearbook as consistent and true magnitudes.

Total industrial value added, and household water withdrawal per capita in both rural and urban areas were taken from province or city Statistical Yearbooks. Finally, the number of employees in accommodation and catering and other services, and area of green land were from the China City Statistical Yearbook^62^. We had no data for Hong Kong, Macao and Taiwan due to limited statistical availability.

## Data Records

Our datasets ‘City-level water withdrawal and scarcity accounts of China’ are publicly available *via* the Figshare repository^63^. A total of 40 data records (subsector water withdrawal, total water withdrawal, criticality ratio inventories, industrial and farming intensity, and industrial output) were assembled in the datasets. Of these:

1. thirty-four are city-level subsector water withdrawal inventory (in the sequence of thirty-four province-level administrative units: we put those cities from the same province into a unit. This also facilitates connection with IO tables) [File ‘China city-level subsector water withdrawal inventory, 2015’];
2. one is city-level total water withdrawal and criticality ratio inventory [File ‘China city-level total water withdrawal and water-scarcity ratio inventory, 2015’];
3. three are city-level subsector water withdrawal inventory in 2012 (in the sequence of three province-level administrative units) [File ‘Subsector water withdrawal of 13 cities from the Beijing-Tianjin-Hebei agglomeration, 2012’].
4. one is city-level subsector industrial and farming water withdrawal intensity dataset (in the sequence of thirty-four province-level administrative units) [File ‘Industrial water withdrawal intensity and Farming irrigation intensity, 2015’].
5. one is city-level subsector industrial output dataset (in the sequence of thirty-four province-level administrative units) [File ‘City-level subsector industrial output dataset, 2015’].

Average water withdrawal per industrial GDP at city level was 50 m^3^ per US$10^3^. From a global perspective this still have a gap compared with the worldwide countries with first-class water use efficiencies averaging 33 m^3^ per US$10^3^ ^64–66^. At the city level, industrial water withdrawal accounted for a mean average of 24% in overall production water-use. This proportion ranged from 0.24% in Hotan (in northwest China), to 35% in megacities like Shanghai (east China), Chongqing (southwest China), Nanjing (mid-east China) and Fuzhou (south-east China), and up to 94.83% in Baishan (northeast China). Industrial water withdrawals were ranked highest in 17% of cities, and in the top two places in 97% of cities among productive water withdrawals.

## Technical Validation

### Uncertainties

For method validation we referred to and used procedures in previous studies^10,67^. Overall, for Case 2 on estimation for cities without water withdrawal statistics, water withdrawal efficiency was a key uncertainty. We conducted sensitivity analyses by replacing these efficiency data with regional efficiency. The regional efficiency data were from provincial and regional Water Resources Bulletins.

Integrated sensitivity of total water withdrawal of cities in Case 2 was 7.8%. It showed differences between replaced total industrial water withdrawal and our original estimations ranged from −13.5% in Xuzhou, Lianyungang, and Huai’an, to 9.5% in Nantong, Zhenjiang and Taizhou. The average difference in absolute value was 7.3%. This result indicates a relatively small difference, and validates the method in Case 2 as a credible estimation of industrial water withdrawal. Similarly, difference ranges were 9.0% for agriculture water withdrawal and 8.0% for service water withdrawal, compared to estimations with regional intensities. We omitted sensitivity tests for environment and ecology water withdrawal due to a lack of comparable data on water withdrawal per sanitary area^68^. There was no uncertainty for Case 1.

Notably, variability of intensity for each city was considered for regionalization of water withdrawal. China’s cities showcased distinctive characteristics in terms of age, size, coverage, population, resource endowment and industrial drivers, and this heterogeneity indicated different economic development levels^69,70^. Hence, we tuned water withdrawal based on local statistics, such as gross water withdrawal in each type, for calibration. This calibration reflected local water status accurately. Even in Case 2, where not all data were available, differences between cities were considered and intensities of economically and demographically similar regions were used to estimate for cities at a similar stage^71^. Thus, our datasets may be more advanced than those in the three previous studies^26,53,72^, which assumed agriculture-, industry-, construction- and service-water withdrawal intensities were identical for every city and even transferred information and data of rich cities to serve for poor cities. For example, because implementation of its WA + framework did not necessarily require local-measured or representative indicators or efficiency data, the previous study results^72^ may suffer from substantial biases. For more methodological validation please refer to detailed discussions in references^10,14,15^.

### Comparisons with other datasets

#### Comparisons with a previous study

In Table 3, we compare gaps in water withdrawal between estimations of a previous study by Zhou *et al*.^24^ and our owns. We chose thirteen Beijing-Tianjin-Hebei cities in 2012, considering these cities to be one of the most significantly water-scarce regions. We additionally estimated 2012 data, using an identical methodology, because Zhou *et al*.^24^ merely provided data before 2013. These data were attached separately and extendedly as time-series datasets.

**Table 3:** A comparison of water withdrawal (100 million m^3^).

City	Farming			Industry			Urban household			Rural household			Total		
	Zhou *et al*.^24^	This study	Gap (%)	Zhou *et al*.^24^	This study	Gap (%)	Zhou *et al*.^24^	This study	Gap (%)	Zhou *et al*.^24^	This study	Gap (%)	Zhou *et al*.^24^	This study	Gap (%)
Baoding	21.13	22.76	−7	1.58	1.18	34	0.83	1.57	−47	2.13	1.88	13	26.25	31.09	−16
Cangzhou	7.75	9.02	−14	0.52	1.08	−51	1.12	0.74	50	0.86	1.14	−25	11.12	13.91	−20
Chengde	6.17	5.71	8	1.42	1.02	40	1.06	0.80	33	0.96	0.88	9	9.89	9.16	8
Handan	11.48	14.02	−18	1.86	1.83	1	2.93	1.17	150	2.38	1.38	72	20.17	20.72	−3
Hengshui	12.27	12.80	−4	0.81	0.69	17	1.25	0.45	177	1.10	0.66	67	16.05	15.77	2
Langfang	6.11	5.72	7	0.84	0.79	6	2.36	0.86	174	0.59	0.88	−33	11.18	10.26	9
Qinhuangdao	5.49	5.27	4	0.98	0.94	4	1.52	0.71	114	1.03	0.70	47	9.54	8.88	7
Shijiazhuang	19.94	20.69	−4	2.81	2.04	38	2.76	2.38	16	2.39	1.85	30	29.34	31.89	−8
Tangshan	16.95	14.32	18	4.84	4.82	0	2.61	2.29	14	2.41	1.68	44	28.63	25.97	10
Xingtai	13.00	13.29	−2	1.71	1.24	38	1.28	0.69	85	1.24	1.12	10	17.75	18.12	−2
Zhangjiakou	7.97	7.40	8	1.21	0.82	47	1.39	0.51	171	0.78	0.85	−7	11.67	10.56	11
Tianjin	7.11	*	−	5.36	5.10	5	7.29	*	−	1.18	*	−	26.24	23.10	14
Beijing	7.16	*	−	4.90	4.90	0	10.14	*	−	0.58	*	−	28.48	35.90	−21
Total	128.27	130.99	−2	28.85	26.45	9	19.10	12.18	57	15.87	13.01	22	246.31	255.33	−4

Notes: i) gaps >10% are highlighted; ii) * means not disclosed in the Bulletin; iii) the GDP deflator was calculated as 1.098 between 2010 and 2012.

Water withdrawals for farming were found to be in close agreement between the two studies. Total water withdrawal calculated by Zhou *et al*.^24^ was 2% lower than that of this study, on average. For industry, rural- and urban- household water withdrawals substantial differences between these two studies were found. For industry, water withdrawal was on average 9% lower for Zhou *et al*.^24^ than for our study. For rural household (defined by Zhou *et al*.^24^ as water withdrawal for livestock and poultry-breeding), water withdrawal was 22% higher than in our study, on average. For urban household (including service in Zhou *et al*.^24^), water withdrawal was 57% higher than in our study. Total water withdrawal was, on average, 4% lower in Zhou *et al*.^24^ than in this study. The reasons for these differences could be twofold: (1) these data are derived from different sources, i.e., city water resources bulletin in the present study, compared to simulations combined with provincial and national water resources bulletin in Zhou *et al*.^24^; and (2) there have been name changes, merging and separation of data between cities in the historical statistics^73^, as summarized in Table 4 for detail.

**Table 4:** Name and boundary change of city in ArcGIS shapefile data of China’s prefectures, compared to Zhou *et al*.^24^ and others.

Type	Code of city	Original	Corrected
Merging	C12	Chaohu (city), Hefei (city), Ma’anshan (city)	Hefei
Separation	C85	Haikou (city)	Haikou, Sanya & Danzhou
	C116	Tahe (county)	Tahe & Great Khingan
Name Change	C27	Pingliang (city)	Baiyin
	C61	Jiangmen (city)	Zhuhai
	C71	Heshan (city)	Liuzhou
	C166	Mudanjiang (city)	Yanbian
	C175	Changzhou (city)	Wuxi
	C218	Guyuan (city)	Pingliang
	C221	Wuzhong (city)	Yinchuan
	C222	Wuzhong (city)	Zhongwei
	C280	Zunyi (city)	Luzhou
	C316	Yunlong (county)	Dali
	C340	Daishan (county)	Zhoushan

Note: ArcMap 10.3.1 version was used. The same coordinate system was applied to all 343 cities.

#### Comparison between scope and method of accounting from different data sources

**Fig. 5:**
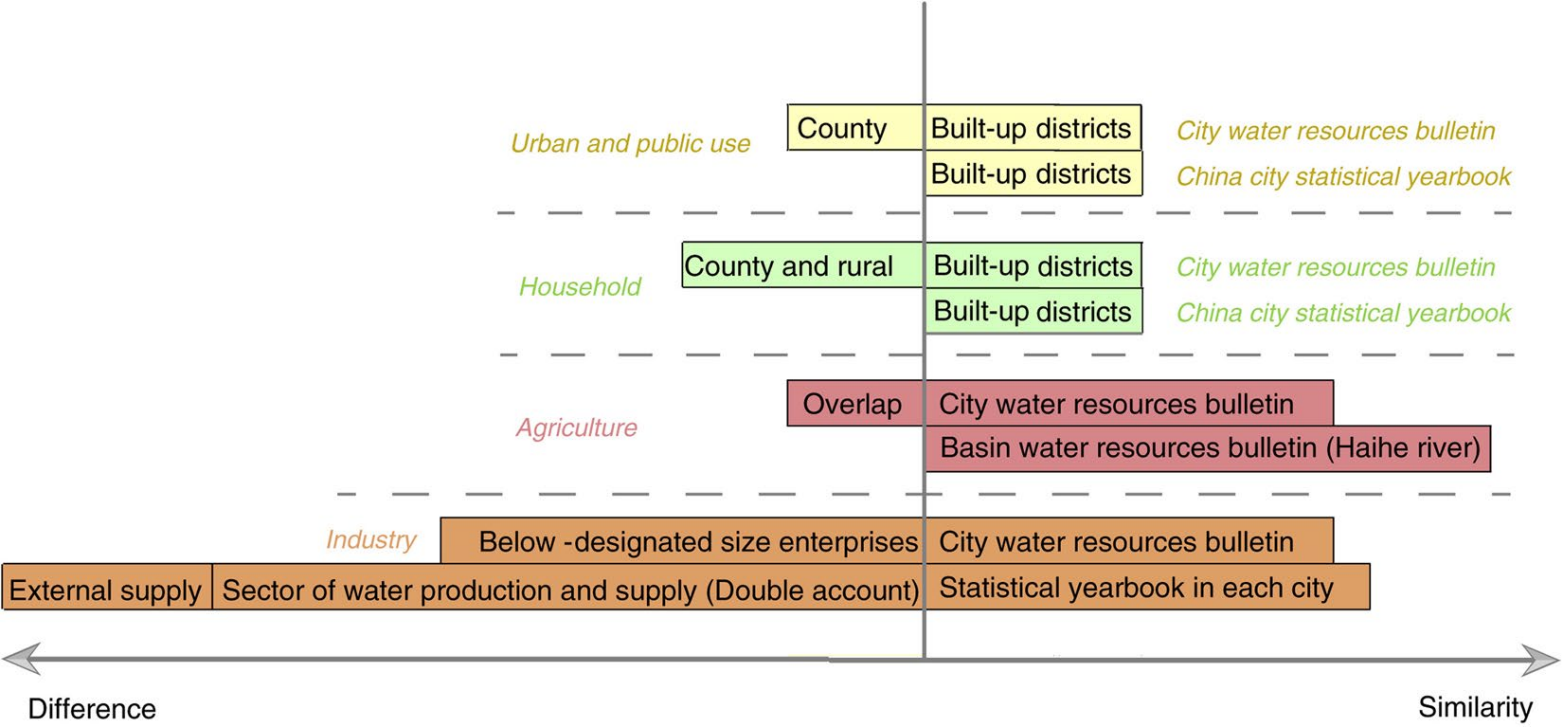
Comparison between different scopes of water-use accounting sectors, from various data sources in China.

For total and subsector data, although they are reported directly in official and public statistical bulletins, different bulletins at different administrative levels (city and province), or from different governing ministries (i.e., *Ministry of Ecology and Environment* (as in this study) and *Ministry of Water Resources* (as in Zhou *et al*.^24^) have inconsistent and even contradictory elements. Thus, to ensure reliable and unbiased data production and, as highlighted by Zhou *et al*.^24^, harmonize official statistics of water withdrawal, we summarized different scopes and accounting methods from various sources in China (Fig. 5). Differences between datasets are summarized to the left of the x-axis, while identical points are on the right. Given different calibres were in-use, only a limited comparison was possible between these datasets^74^.

First, basin-level Water Resources Bulletins also provide the same sectors of data as city-level bulletins. For example, Water Resources Bulletins for the Haihe Basin have data for the cities of Beijing, Tianjin, and Hebei. However, data on agriculture and household water withdrawals in Beijing are larger and smaller, respectively, than those in the city-level Water Resources Bulletin by approximately 60 million m^3^. These differences could be attributed to adjustments since 2012, for example water withdrawal for livestock has been transferred from household water withdrawal to agricultural water withdrawal in the city bulletins. Thus, these two sources may contain overlaps. In addition, regarding household water withdrawal, data from the China City Statistical Yearbook only cover built-up districts, while City Water Resources Bulletins cover all built-up districts, counties, and rural areas. In this case, we regard data from city-level Water Resources Bulletins as a benchmark to obtain a consistent data source. This helps ensure datasets used in this study are the most consistent and robust based on available-to-date water statistics in China.

Second, a number of statistical yearbooks at the city level also provide subsector industrial water statistics. Nevertheless, data availability is quite limited. For example, in 2012 there were only 59 cities in China that had subsector industrial water withdrawal data. Moreover, total industrial water withdrawal statistics in yearbooks exhibit discrepancies and inconsistencies with those provided in water resources bulletins. Possible reasons may be that (1) bulletin statistics incorporate water withdrawal of companies below a designated size, while yearbook statistics usually cover only companies above a designated size. (2) yearbook statistics include water withdrawal for external supply, while bulletin statistics omit this information. (3) water withdrawal in the water production and supply sub-sector is regarded as zero in this study to avoid duplicate accounting: we consider interactions between the water production sub-sector and other sub-sectors (as stressed in a previous study^23^). This sub-sector is comprised of tap water production and supply, and sewage treatment. The former supplies water to other sub-sectors, and the latter uses little additional water^75^. However, the statistical yearbook may double-account water withdrawal in the water production and supply sub-sector. Similarly, in post-2009 yearbook statistics, water withdrawal data have excluded cooling water from rivers, lakes, and seas, while bulletin data retains it. In addition, data from statistical yearbooks are incomparable with data from previous years since the National Bureau of Statistics has adjusted its investigation methods in terms of calibres and periods. For example, according to the China Statistical Yearbook, data to 2008 covers all state-owned and above-designated size companies. After 2008, coverage of all industries above the designated size is adopted, referring to companies with annual revenue >US$0.73 million between 2008 and 2010, and >US$2.93 million since 2011 and to date in 2023. Given these facts, using yearbook data of subsector industrial water statistics may be problematic due to internal inconsistencies, and introduce additional uncertainty when compared with water withdrawal of other types.

Third, attention needs to be paid to discrepancies and inconsistences between water supply and water use in China’s statistics. While these two are equal in a number of cities (for example in Tianjin), water supply is not equal to water use in other cities. This discrepancy could be attributed to loss of water in raw water storage and transfer facilities such as impoundment reservoirs or aqueducts, or in the water treatment and distribution system such as waterworks, storage reservoirs and pipelines. There is also some systematic uncertainty in local statistics^74^, for example, enterprises will be removed from China’s water statistics if they cease operating for a period of time or stop trading altogether; however, this status is determined and reported by the enterprises themselves. Thus, water withdrawal compiled and estimated in this study may be under-estimated and suffer from downward bias.

Finally for service water withdrawal, considering most services mainly use tap water^76,77^ and in order to find an accurate and rigorous driving force (size indicator), we also considered proportions of input from water production and supply into each service in the IO tables. We compared this method to the method using subsector-employee number and found results of IO input were more reasonable. In this calculation, imports of each sub-sector were excluded from the original IO table to obtain domestic input; this could depict local economic interactions more reasonably. However, no more than 20 cities have IO tables, hence this IO method would only become more preferable when more city level IO tables are available.

### Limitations and future work

As with all studies of this type there are some limitations: firstly, the original data in the basin- or city-level statistical yearbooks are either not available or insufficient to support a systematic comparison at this stage. In other words, they could not provide sufficient content to merit a systematic comparison for publication. We would like to conduct this as a part of our future work, which may also include updates to these datasets, fuller accounting and comparison of datasets, and additional information to aid reuse.

Secondly, we have not considered water amounts from water transfer projects, such as the South-to-North water diversion projects in the north China plain^78–80^. Data from these projects are generally not available, and we have only managed to obtain some for the middle route. We hope to supplement this in future work. This study would also provide a foundation or reference for process-based and refined work, i.e., subsector techniques.

## Usage Notes

Potential uses of the datasets are: (1) these data could be used directly in Input-Output (IO) models (combined with IO tables at the megacity, provincial, and national levels), consumption-based accounting and structural decomposition analyses. Such analyses may help gain in-depth insights into subsector water-saving priorities, industrial transfers and market restrictions towards development of a water-saving society^33^ in China, and facilitation of relevant UN Sustainable Development Goals around universal access to safe and clean water (6.2.1, 6.4.1, 6.4.2) by 2030. (2) Data could be put to practical use in municipal design and government planning. For example, given the fact that water-use quotas for sectors in many cities suffer from large uncertainties, data could be used in verification for industrial organizations, and business case studies for Nike brand promotion (http://wwwen.ipe.org.cn/GreenSupplyChain/BrandStoryDetail.aspx?id=57). (3) These inventories would facilitate regional water-status education and training. For example, by making comparisons across whole cities and economic-sectors, educators and trainers could target subsector water saving and improve efficiency in specific (minor) sectors and cities, i.e., identification of low-efficiency sub-sectors and users in water-stressed megacities from China^81,82^ (Table 5) including Shenzhen, Beijing, Shanghai, Chengdu, Tianjin, Qingdao, Suzhou, Shijiazhuang, Zhengzhou, Guangzhou, Xi’an, Wuhan, Nanjing and Linyi cities etc. which contained more than 10 million inhabitants in 2022. The 2015 data should also be representative for time-series information since China’s precipitation (and water availability) was 2.8% (0.9%) higher than, but close to, its average through multiple years (1957–2022, with statistics)^56^.

**Table 5:** Representative water-scarce Chinese megacities and their classifications.

Water scarcity class	Representative megacities
Extreme water scarcity (criticality ratio >100%)	Qingdao, Handan, Shijiazhuang, Tianjin, Shenyang, Zhengzhou, Jining, Xuzhou, Beijing, Tangshan, Jinan, Baoding, Dalian, Suzhou, Weifang, Shanghai, Chengdu, Shenzhen, Heze, Yangzhou, etc.;
High water scarcity (40% <criticality ratio <100%)	Zhoukou, Changchun, Shangqiu, Nanjing, Cangzhou, Luoyang, Xi’an, Linyi, Guangzhou, Nantong, Wuhan, Yancheng, Harbin, Nanyang, Fuyang, Xiamen, Wuxi, etc.

## Data Availability

No custom code was used.
